# Positron emission tomography tracers for synucleinopathies

**DOI:** 10.1186/s13024-024-00787-9

**Published:** 2025-01-05

**Authors:** Jie Xiang, Zhentao Zhang, Shengxi Wu, Keqiang Ye

**Affiliations:** 1 Department of Neurobiology, Fourth Military Medical University, Xi’an, 710032 China; 2 Department of Neurology, Renmin Hospital of Wuhan University, Wuhan, 430060 China; 3 Faculty of Life and Health Sciences, Shenzhen University of Advanced Technology (SUAT), Shenzhen, China; 4 Brain Cognition and Brain Disease Institute (BCBDI), Shenzhen Institute of Advanced Technology (SIAT), Chinese Academy of Sciences, Shenzhen, Guangdong 518055 China

**Keywords:** α-synuclein, Positron emission tomography imaging, Tracer development

## Abstract

Synucleinopathies, such as Parkinson’s disease, dementia with Lewy bodies, and multiple system atrophy, are characterized by the aggregation of α-synuclein. Variations in brain distribution allow for differentiation among these diseases and facilitate precise clinical diagnosis. However, distinguishing between synucleinopathies and Parkinsonism with tauopathies poses a challenge, significantly impacting clinical drug development. Therefore, molecular imaging is crucial for synucleinopathies, particularly for clinical diagnosis, assessment of drug efficacy, and disease surveillance. In recent years, advances in molecular imaging have led to rapid development of α-synuclein-specific tracers for positron emission tomography (PET), most of which are still in pre-clinical stages. Interestingly, some of these tracers share similar compound skeletal structures and are currently undergoing optimization for clinical application. Despite this progress, there remain challenges in developing α-synuclein tracers. This review summarizes recent findings on promising PET tracers and discusses representative compounds’ characteristics while offering suggestions for further research orientation.

**Supplementary Information:**

The online version contains supplementary material available at 10.1186/s13024-024-00787-9.

## Background

Synucleinopathies are a group of neurodegenerative diseases characterized by abnormal α-synuclein (α-Syn) deposition in neurons, nerve fibers, or glial cells. Parkinson’s disease (PD), dementia with Lewy bodies (DLB), and multiple system atrophy (MSA) are the most prevalent synucleinopathies [1]. Parkinson’s disease is the most widespread progressive neurodegenerative movement disorder among these. Although these diseases can be managed with medication to alleviate symptoms, there is currently no cure. Currently, approximately 7 to 10 million people worldwide are affected by PD. In China, the number of PD patients is expected to increase to 4.94 million in 2030, accounting for half of all PD patients worldwide [2]. The yearly expense for each PD patient in China averages $3,225.94, with direct costs being $2,503.46 and indirect costs amounting to $722.48 [3].

Common motor symptoms of parkinsonism include resting tremor, rigidity, bradykinesia, and gait freezing. However, in the early stage of the disease, PD patients often present with a series of nonmotor symptoms, such as constipation, depression, and sleep disorders. The disease can also progress to cognitive impairment and psychosis in the late stage. In patients with dementia with Lewy bodies (DLB), cognitive dysfunction is a critical diagnostic feature [4]. Patients with DLB may exhibit a wide range of impairments, including cognitive issues, neuropsychiatric symptoms, sleep disturbances, motor difficulties, and autonomic dysfunction [5]. Visual perception is also affected in DLB patients. Visual hallucinations are often early symptoms of the condition. MSA usually appears as either parkinsonism (MSA-P) or cerebellar ataxia (MSA-C), with accompanying autonomic dysfunctions such as urinary incontinence, orthostatic hypotension, and pyramidal symptoms. The rates of disease progression vary among different synucleinopathies [5].

The distribution of α-Syn aggregates is different in synucleinopathies. In PD patients, aggregated α-Syn is distributed mainly in the midbrain, especially in the substantia nigra pars compacta, and in a broader range of areas, such as the limbic and cortical areas, in the late stage of the disease. The early stage of DLB involves the cerebral cortex; the limbic system and hippocampus are affected later. In MSA patients, α-Syn accumulation is detected mainly in the olivo-pontine cerebellum, substantia nigra, striatum, and autonomic nervous system. α-Syn inclusions are also present in different cell types. In PD and DLB, the Lewy bodies (LBs) and Lewy axons (LNs) of neurons are found mainly in the cytoplasm and axons of neurons. In MSA, however, glial cytoplasmic inclusions (GCIs) are present in the cytoplasm of oligodendrocytes [6]. LBs and GCIs have different morphologies and possibly different compositions. LBs are more rounded and denser than GCIs under fluorescent confocal observation [7]. Interestingly, pathological α-Syn aggregates have been found in peripheral organs such as the liver, heart, gastrointestinal tract, skin, and mucosal tissues [8]. The presence of pathological α-Syn in peripheral organs, the interaction between body fluids and the autonomic nervous system, the involvement of BBB receptors, and the impact of peripheral fibrillization microenvironment (FME) on α-Syn properties all suggest that peripheral organs may be the origin of PD pathology and even play a role in initiating α-Syn pathology. Although the clinical and phenotypic differences between synucleinopathies are well known, the mechanisms by which these pathological changes occur and their associations with the clinical phenotype are still unclear.

Although the deposition of α-Syn is a pathological hallmark of synucleinopathies, the lack of methods for detecting α-Syn deposition in vivo limits accurate clinical diagnosis. It hinders the development of new treatment methods for these diseases. Molecular imaging with positron emission tomography (PET) can potentially reveal the pathogenesis of brain diseases by locating and quantifying drug targets, monitoring therapeutic effects, or imaging on a molecular basis. Several studies have shown the importance of imaging for including PD patients in clinical trials [9]. Dopaminergic pathway imaging, like DAT (dopamine transporter) imaging, can be used to monitor the efficacy of investigational disease-modifying drugs in PD. However, it is difficult to differentiate between MSA and PD patients. Moreover, some studies suggest that DAT imaging may initially be standard in some DLB subtypes (∼10% of cases), with possibly a different severity or spread of α-synuclein pathology [10, 11]. Therefore, imaging α-Syn is highly important for monitoring disease progression and early diagnosis, which may provide evidence for the effectiveness of drug treatment. Thus, imaging α-Syn deposition represents a breakthrough in diagnosing PD. This review provides a detailed summary of the currently developed potential radiotracers that target α-Syn. The prospects of potential new diagnostic tracers are discussed.

## Targeting α-Syn

### α-Syn

The 140-amino acid protein α-Syn is expressed at varying levels in the brain, peripheral nerves, and other tissues. It comprises an N-terminal amphipathic helix (amino acids 1–60) that interacts with lipid membranes and determines fiber formation [12]. The non-amyloid component (NAC), located in the central core region (amino acids 61–95), promotes aggregation and amyloid formation by facilitating protein-protein interactions [13, 14]. Truncation of its C-terminal region increases fiber formation. Additionally, the N-terminal region harbors familial mutations [15]. Physiologically, full-length α-Syn primarily exists as an unfolded monomer and plays a role in regulating exocytosis and neurotransmitter release as a presynaptic protein. Nevertheless, knockdown of α-Syn results in only mild phenotypic abnormalities despite its abundance in cytosolic proteins [16]. Currently, most interest is focused on its role in synucleinopathies. Missense mutations in the *SNCA* gene can be found in inherited PD patients, thus confirming the link between α-Syn and PD [17]. Nonetheless, owing to the lack of a direct correlation between α-Syn deposition and neuronal death [18], the mechanism of action of α-Syn in PD progression remains unclear. In fact, at high concentrations of monomers or in the presence of interferers (for example, in the presence of oscillations or beads), α-Syn first forms various oligomers that slowly convert into aggregates containing parallel β-sheet structures [14]. The growth of α-Syn aggregates can be accelerated by recruiting and transforming monomers to form amyloid fibrils with unique crossover structures. Recent studies examining α-Syn aggregates in the brain and biological fluids have also shown that α-Syn aggregates can be essential biomarkers for detecting PD and other synucleinopathies [19, 20]. The α-Syn seed amplification assay (αSyn-SAA) accurately detects all PD samples as positive, achieving a sensitivity of 100%, and distinguishes the PD group from healthy controls with a specificity of 70.8% (*p* < 0.0001) as well as from tauopathies like CBD (71% specificity) and PSP (75% specificity). The α-Syn-SAA can also detect nearly all MSA samples as positive for α-Syn aggregation, with a sensitivity of 92.6% [21]. Another cohort study reports a sensitivity of 87.7% for Parkinson’s disease and a specificity of 96.3% for healthy controls. The sensitivity of α-Syn SAA in sporadic PD with a typical olfactory deficit is 98.6%. In prodromal and at-risk groups, 86% of individuals with RBD or hyposmia tested positive for α-synuclein SAA (16 out of 18 with hyposmia and 28 out of 33 with RBD) [22]. Goedert M and his colleagues demonstrated that PD, PDD, and DLB consist of a single protofilament (Lewy fold), which is significantly distinct from the protofilaments found in MSA [13]. However, it remains unclear why LBD or MSA have structurally different subtypes. Establishing links between seeding properties, disease duration, and pathologic progression would advance structure-based early diagnosis. Additionally, creating PET probes that target the disease-specific filament core is crucial for distinguishing between LBD and MSA in the early stages of the disease [23].

### Synucleinopathies

Intraneuronal inclusion bodies with α-Syn as the main component are found in PD and DLB patients. Compared with those in PD patients, protein aggregates in DLB patients are located predominantly in the cerebral cortex [19]. In MSA patients, α-Syn is also the primary filamentous element in glial and neuronal cytoplasmic inclusions [24]. Unlike inclusion bodies in PD and DLB, MSA features filamentous components in neurons and oligodendrocytes [19, 25–27]. Structural variations are observed in α-Syn fibrils from patients with various synucleinopathies. PD and MSA fibers show flat and twisted forms, while DLB fibers display untwisted columnar shapes [28]. The exact role of α-Syn in PD, DLB, and MSA remains unclear. However, α-Syn oligomeric forms are suspected to induce neurotoxicity by interfering with cellular homeostasis and affecting intracellular components, which results in mitochondrial toxicity, increased synaptic, inflammatory responses, and endoplasmic dysfunction [29–33]. Additionally, it has been reported that α-Syn leads to the release of free radicals, which contribute to neurodegeneration. Finally, neurons containing α-Syn inclusion bodies have impaired DNA repair function [31, 34, 35].

### Challenges in the development of α-Syn radiotracers

The development of PET tracers for α-Syn is more complicated than for other amyloid proteins, such as amyloid-β (Aβ) or Tau. This is mainly because the concentration of α-Syn aggregates in the brain is much lower, requiring tracers with very high affinity, likely in the nanomolar range [6, 7, 36]. Thus, PET tracers for α-Syn need higher selectivity than those for Aβ and Tau because of their coexistence and colocalization with Aβ and Tau fibers. For instance, Aβ deposition is present in about 85% of DLB cases, while Tau pathology is found in roughly 30% [6, 37–39].

The formation of Aβ or Tau fibrils results in β-fold sheets that are structurally similar, posing challenges for developing ligands that selectively bind to α-Syn. Additionally, unlike Aβ inclusions, α-Syn inclusions are predominantly inside cells. Therefore, any effective α-Syn PET tracer must be capable of crossing both cell membranes and the blood-brain barrier (BBB) [37, 40]. While this is generally not considered a significant obstacle, it may still present some issues. For example, the D2/3 PET tracer [^11^C] raclopride can cross the BBB but not the cell membrane [41]. Furthermore, various post-transcriptional modifications of α-Syn in vivo may result in multiple substructures that cannot be recognized by tracers designed for unmodified structures [37, 40].

Amyloid tracer development encounters difficulties with standard strategies that are typically used for more conventional targets like receptors and enzymes. These strategies require a well-defined binding pocket to apply directly to protein-based structures. The fundamental mechanisms of binding are not well comprehended, and attempts to pinpoint binding sites or uncover kinetic and thermodynamic parameters of ligand binding through computational or other methods are still in their infancy [32, 42, 43].

It has been suggested that the differences in the structures of α-Syn between aggregates found in patient brains and fibers formed in vitro could contribute to the challenges in developing new α-Syn PET tracers for clinical studies [44]. Previous research has indicated that there is structural diversity in brain-derived substances from patients with PD and MSA [44, 45]. There is still uncertainty about how α-synuclein is linked to different cellular pathologies like Lewy bodies and glial cytoplasmic inclusions, as well as what factors make certain neuroanatomical areas and cell types more vulnerable. Growing evidence indicates that α-Syn species from Lewy body disease and MSA are unique ‘strains’ with varying seeding characteristics [46]. α-Syn can self-assemble and form multiple conformational polymorphs, much like prions, in both in vitro and in vivo conditions. Derived from the same precursor protein, these α-Syn polymorphs could have distinct biochemical characteristics and might lead to different pathological phenotypes when introduced into animal models [47]. Moreover, changes in the local or global microenvironment significantly affect fibril formation. A single amino acid alteration can induce polymorphism due to distinct site-specific conformational dynamics, as seen in the wild type and E46K, A30P, and A53T fibrils [48]. Noticeably, fibril polymorphs formed by PD mutants have different morphology and secondary structures than those of wild-type protein [49]. Cryo-EM analysis of fibrils formed by mutants showed their flexibility regarding twists, the number of interacting protofilaments, packing arrangement, secondary structure elements, and quaternary shape [50]. The impact of these variations on how potential ligands bind to α-Syn remains unclear. The formation of α-Syn deposits in animal models raises questions about their structural similarity to those found in humans and their possible use for screening selective ligands. The choice of animal model is crucial for the outcome of these experiments, with recent research focusing on disease models that can simulate intracellular inclusions similar to those observed in humans. While wild-type healthy animals like mice, rats, and nonhuman primates are typically used in vivo animal testing, there is a growing preference for transgenic rodent models as they better mimic human brain aggregates.

### α-Syn PET tracer development

**Fig. 1:**
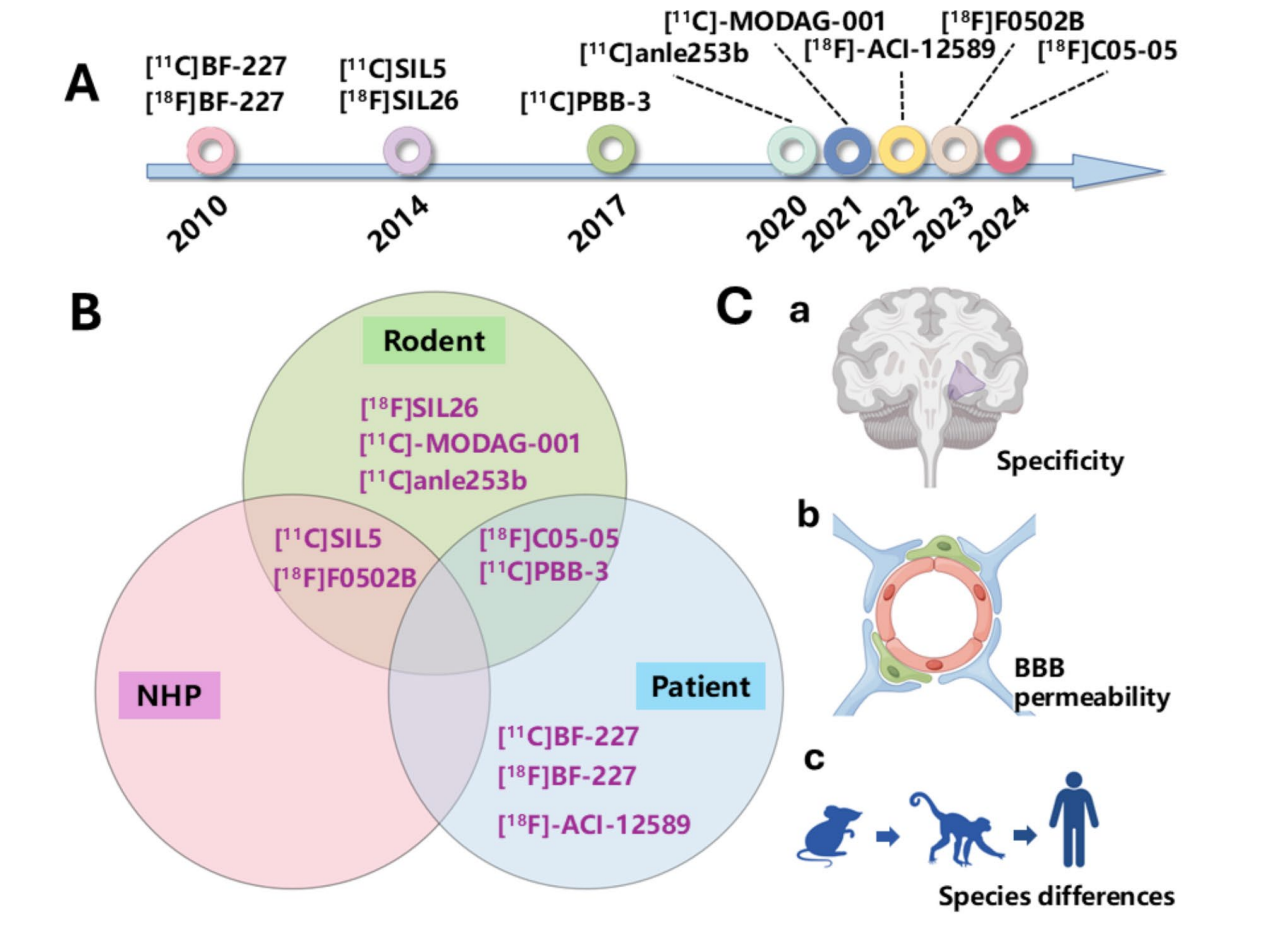
Representative α-Synuclein PET imaging. (**A**), the timeline of representative PET tracers’ development. (**B**), the PET imaging objectives used in different PET tracers. NHP: non-human primates. (**C**), the major challenges of developing an ideal α-Synuclein PET tracer. BBB, blood-brain barrier

In recent decades, various compounds that target α-Syn have been developed. These compounds have been created via multiple methods, such as rational drug design and high-throughput screening. This portion is dedicated to selecting these structures, evaluating their potential as PET tracers specific to α-Syn., and thoroughly evaluating their biological effects in vitro and in vivo. From a broader perspective, the studies mentioned above indicate the possibility of selectively binding α-Syn and imaging it in postmortem human tissues. In subsequent studies, these ligands are considered promising starting points for developing other α-Syn-selective ligands (Fig. 1**).**

#### Phenothiazine analogs (SILs)

Phenothiazine analogs (SILs) have been extensively researched as potential selective α-Syn PET ligands. These compounds were developed through the application of rational tracer design principles. For instance, structurally similar diarylimides have demonstrated neuroprotective effects in PD models because of their antioxidant characteristics [51]. In this structural group, several phenothiazines prevent the aggregation of insoluble α-Syn filaments with IC_50_ values in the low micromolar range [51–53]. A dimethoxy-substituted phenothiazine analog with a K_d_ near 120 nM is the first promising structure found in this group. The substitution of a methoxy group with cyano or amine results in a reduction of affinity by over 3 to 5-fold. Conversely, substitution with a nitro group (SIL5) increases the affinity by approximately 4-fold [54]. In subsequent studies, further attempts to modify this position with other substituents to enhance the affinity were unsuccessful [55]. SAR studies focused on the methoxy group of SIL5, with _i_K_d_ values ranging from 32.1 nM to 83.3 nM. Based on these discoveries, Bagchi et al. used iodine-125 to radiolabel SIL23. They assessed its affinity for Aβ1–42 and Tau fibrils and its binding properties to LBs and LNs in human PD brains [56]. SIL23 demonstrated a K_d_ value of 148 nM on α-Syn fibers, revealing similar affinity in human tissues. However, these values are approximately half of those determined by the fluorescent thioflavin T (ThT) competition assay, indicating binding differences between the radiolabeling and competition assays. Besides, its Aβ and Tau fibers selectivity are also limited. Therefore, SIL23 is unsuitable for developing PET α-Syn tracers.

#### BF-227

BF-227 was initially created as a PET tracer to detect amyloid plaques in individuals with Alzheimer’s Disease [57]. However, during the biological assessment, BF-227 also demonstrated a 10-fold lower affinity for α-Syn fibers (K_d_ 9.63 nM) than for Aβ1–42 fibers (K_d_ 1.31 nM). These findings led to further evaluation of BF-227 in human brain tissue. There are inconsistent findings regarding BF-227’s capacity to identify α-Syn in human brain tissue [58–60]. For example, Fodero-Tavoletti et al. found that BF-227 binds to α-Syn in patients with PD but not those with DLB [58]. The following research assessed the binding properties of BF-227 in postmortem tissues from individuals with PD and DLB, demonstrating its capability to identify α-Syn fibers in all cases [61].

A PET study on MSA patients showed that [^11^C]BF-227 effectively visualizes α-Syn deposits in these individuals [62]. However, Verdurand et al. questioned the binding of BF-227 to pathological α-Syn forms [63]. They performed autoradiography studies on brain sections from MSA and control patients, targeting the medulla oblongata to examine BF-227 binding due to its role in MSA progression without Aβ pathology [60]. [^18^F]BF-227 could not identify α-Syn inclusion bodies compared to healthy controls, suggesting that BF-227 might not serve as an α-Syn PET ligand.

#### Anle138b

DPP, or 3,5-Diphenylpyrazole, is a promising small-molecule structure for detecting aggregated α-Syn inclusions. The fluorescence spectra indicated that compounds based on DDP hindered the α-Syn aggregation in vivo by binding with α-Syn fibers. The most encouraging candidate, anle138b, was recognized as an oligomer modulator [64]. Notably, Wagner et al.. reported that anle138b exclusively binds to abnormal prion proteins and α-Syn aggregates in vivo rather than native monomers of α-Syn [64]. Additionally, DPP derivatives directly interact with α-Syn fibers in vitro [65].

Moreover, Anle138b reduced α-Syn oligomer formation by 77% in various animal PD models and slowed pathology progression. Anle138b can be taken orally and crosses the blood-brain barrier without causing apparent toxicity with long-term use. Its intrinsic fluorescence was utilized to verify the binding properties with α-Syn [65]. Anle138b’s fluorescence intensity rose by more than 30 times upon adding aggregated α-Syn, while it did not change with monomeric α-Syn. [^11^C] anle253b was administered to healthy rats and was eliminated entirely from the body within 75 min, indicating low brain absorption with unusual brain kinetics. The authors suggested that a high logP might be responsible for this observation. However, no radioactive metabolites were detected via radioactive HPLC [66]. And the affinity and selectivity of DPP derivatives have not been examined in human tissues.

Another compound, MODAG-001 (clogP = 3.85), with reduced lipophilicity, has recently been discovered from this group of compounds. MODAG-001 showed a strong affinity for α-Syn fibers with a K_d_ of 0.6 ± 0.1 nM, and it moderately bound to Tau protein with a K_d_ of 19 ± 6.4 nM and Aβ fibers with a K_d_ of 20 ± 10 nM. [^11^C] MODAG-001 demonstrated effective BBB permeability in mice and successfully visualized fibril seeding in the rat striatum via PET imaging. However, [^11^C]MODAG-001 failure to identify aggregated α-Syn in DLB patient brain sections highlights the necessity for further investigation [67].

#### ^18^F-ACI-12589

^18^F-ACI-12589 is a novel tracer developed by AC Immune [68]. Initial PET scans using this tracer revealed uptake in the cerebellar white matter of individuals with MSA. The tracer binds to α-Syn fibrils but not other amyloids commonly associated with Lewy bodies, such as Aβ and Tau. However, PET signals are not detectable in the brains of individuals with other synucleinopathies, including PD and DLB. The reasons for the limited effectiveness of this tracer in these cases are currently unclear. The conformation of α-Syn aggregates may also play a role, as these fibrils are known to adopt distinct shapes in different synucleinopathies. Researchers reported that the dissociation constant for the tracer was 8 to 30 nM for α-Syn aggregates in tissue slices and brain homogenates from various synucleinopathies. Interestingly, they reported that the K_d_ value for α-Syn in the frontal cortex of PD patients is 17 nM, whereas it is 30 nM in the cerebellum of MSA patients. Despite its lower affinity, it still functions effectively in MSA. Efforts are ongoing to optimize the compound for PD or other synucleinopathies.

#### PBB3 and derivatives

[^11^C] PBB-3 was originally created to visualize Tau aggregates, with a binding affinity of 1.8 nM in transgenic mice’s neocortical and hippocampal areas. Koga et al. conducted preclinical evaluations to test its α-Syn binding ability, revealing that PBB3 binds to α-Syn at elevated concentrations (32.3 µM), as evidenced by double immunofluorescence staining. At lower concentrations (10 nM), [^11^C]PBB-3 was only bound to high-density GCIs in MSA patients and did not show specific binding in patients with DLB [69].

The compound C05-01, which is similar to PBB3, exhibits a K_d_ value of 3.5 nM for brain homogenates and 25 nM for α-Syn fibers. C05-01 cells were evaluated via fluorescence microscopy and tissue microarray (TMA) methods, which effectively analyze pathological tissues in a high-throughput manner. The TMA utilizes paraffin blocks and allows multiple tissue cores to be measured without consuming the entire tissue block. Additionally, it supports the analysis of tissues from various patients and assists in histological techniques such as immunohistochemistry, immunofluorescence, and histological staining. The binding properties of C05-01 to recombinant α-Syn fibers were determined through saturation and competitive binding experiments, revealing an affinity of 25 to 30 nM. Research using brain homogenates from the DLB amygdala demonstrated different affinities than those documented in earlier studies [70]. [^11^C] PBB3 shows a moderate binding affinity with a K_d_ of 58 nM, whereas the unlabeled C05-01 exhibits a high binding affinity with a K_d_ of 3.5 nM. These results indicate possible variations in the tertiary structure between fibrils and brain homogenates.

A recent study by Endo and Maiko Ono revealed that C05-05 was created by replacing a double bond with a triple bond in a hydrocarbon chain connecting two aromatic rings [71]. The modified compound firmly bound to LBs in the amygdala of patients with DLB. However, it is also bound to neurofibrillary tangles and amyloid plaques in the middle frontal gyrus tissue of individuals with Alzheimer’s disease. In nonhuman primates, ^18^F-C05-05 accumulates in the brain regions containing dense α-Syn aggregates, such as the caudate nucleus, putamen, and substantia nigra. In individuals with PD or DLB, radioactivity remained high in the midbrain after two hours and was correlated with motor impairment severity. Additionally, ^18^F-C05-05 is bound to MSA patients’ putamen and middle cerebellar peduncle. These findings indicate that C05-05 interacts with α-Syn aggregates across different synucleinopathies. Researchers reported that C05-05 has a high affinity for binding to LBs (K_d_ value of 1 nM) but exhibits slow brain uptake and rapid metabolism. Furthermore, it displays less selectivity for binding to tangles and Lewy bodies, posing challenges for distinguishing between synucleinopathies and tauopathies.

#### F0502B

F0502B, a derivative of benzothiazole-vinylphenol, exhibited a K_d_ of 4.26 to 7.32 nM for α-Syn deposition in PD brains [72]. The cryo-electron microscopy structure of α-Syn fibrils in complex with F0502B reveals additional atomic details of the interaction between F0502B and α-Syn fibrils. Two significant densities were observed in the deep groove of each protofilament of the complex fibrils, indicating that these densities likely correspond to the F0502B ligand. F0502B is positioned within a deep cavity on the surface of α-Syn fibers, with its phenol head internally located and its fluorine tail pointing outward. The alignment of F0502B to the α-Syn layer occurs at a 1:1 ratio, forming a robust binding network along the fiber axis. ^18^F-labeled F0502B was utilized in binding assays with recombinant proteins and patient-derived α-Syn, Aβ, and Tau. The K_d_ value for the binding of ^18^F-F0502B to α-Syn was found to be 10.97 nM, which is significantly lower than its affinity for Aβ (K_d_: 109.2 nM) and Tau (K_d_: 120.5 nM). Furthermore, the binding affinity of a-Syn fibers from patients with DLB is even more significant, with K_d_ values of 3.68 and 6.23 nM, respectively. ^18^F-F0502B selectively binds to LBs in the striatum and substantia nigra of PD patients but does not effectively detect amyloid plaques or neurofibrillary tangles (NFTs) in AD patients. These findings suggest that ^18^F-F0502B strongly binds to α-Syn fibers in brain tissue.

The study also revealed that F0502B specifically binds to α-Syn aggregates and could be a PET tracer for diagnosing synucleinopathy. Nevertheless, further refinement may be necessary to improve its brain uptake further and decrease its metabolism rate before this compound can be clinically used to diagnose PD. However, this study has some limitations. While F0502B interacts with a-Syn aggregates in postmortem brain sections, it remains uncertain whether it would be beneficial for imaging a-Syn aggregates in patients. Although specific binding to α-Syn fibers has been demonstrated, considering the relatively lower brain uptake and quick drug metabolism rate, it is essential to validate its potential off-target effects in PD patients. We are conducting clinical trials to verify its applications and working on chemical structure optimization to meet the unmet clinical need.

#### ^124^I or ^89^Zr-labeled α-synuclein antibody

Roshanbin et al. showed specific uptake of an ^124^I-labeled antibody fused to two single chain fragment variables from 8D3 (mAb-(scFv8D3)_2_) in a mouse model with striatal α-Syn deposits [73]. Although mechanisms that allow antibodies to penetrate cells and target intracellular α-Syn deposits via transferrin receptors on neurons have been described, no specific uptake was noted in transgenic models with mainly intraneuronal deposits present (L61 and A30P mice) [74]. The rapid clearance of ^124^I from cells after antibodies are internalized and degraded suggests it may not be suitable due to its non-residualizing properties. Notably, a recent study utilized ^89^Zr for labeling synuclein antibody [75]. They created the bispecific Adu-8D3 BBB-shuttle using a transferrin binding unit, a single chain Fab fragment derived from 8D3 (scFab8D3). Although PET images at 168 h p.i. showed uptake at the PFF deposition site for [^89^Zr]-HLu-3-scFab8D3, it was not consistent in all mice. Furthermore, there was no significant difference in uptake for [^89^Zr]-HLu-3-scFab8D3 in PFF-injected F28Tg mice between the cortex and reference region. The rapid clearance of these BBB-shuttles from the blood can be attributed to the interaction between 8D3 and mTfR1 in the periphery [76]. These studies indicated potential limitations when using certain tracers related to α-Syn deposition for imaging purposes.

## Conclusions

Despite significant advances in the management of PD, it remains a substantial cause of disability. Given the apparent role of α-Syn in all stages of disease pathology, diagnostic studies targeting α-Syn are especially critical. Previous studies have confirmed the binding properties of specific chemical structures for α-Syn fiber imaging. It is crucial to conduct control experiments to eliminate the possibility of binding to other fibrous structures, such as Aβ or Tau, particularly in the human brains due to the co-pathologies. Recent studies on synuclein PET tracers have revealed structural similarities, with unsaturated hydrocarbons binding to benzothiazole and benzene ring structure derivatives, suggesting their potential for higher affinity binding with α-Syn [72, 77]. However, many chemicals exhibit lower brain uptake and increased background in vivo PET imaging, highlighting the requirement for further efforts to enhance brain uptake in vivo. Clinical trials are needed to assess whether chemical structures can be used to image α-Syn fibers in the human brain accurately. Pathologically, PD is characterized by the deposition of α-Syn aggregates and the degeneration of nigrostriatal dopaminergic neurons. PET scans have been developed to assess the loss of the nigrostriatal dopaminergic pathway. However, the development of α-Syn PET tracers is still preliminary and needs further research. The varying conformations of α-Syn in MSA, DLB, and PD may also complicate the interpretation and impact the results. Clearly, further optimization is required in order to determine the proper imaging methods for the early and accurate diagnosis of synucleinopathies.

## Electronic Supplementary Material

Below is the link to the electronic supplementary material.

Supplementary Material 1

## Data Availability

Not applicable.
