# Supplementary material for: Positron emission tomography tracers for synucleinopathies
Source: Mol Neurodegener. 2025 Jan 5;20:1. doi: 10.1186/s13024-024-00787-9 (PMC11702101; doi:10.1186/s13024-024-00787-9)
Supplement: Supplementary file 1 — Supplementary Material 1 [file 13024_2024_787_MOESM1_ESM.docx]

Novemember 30^th^, 2024

Dr. Owen Ross, Ph.D.

Molecular Neurodegeneration

Dear Dr. Ross,

Thank you for monitoring our manuscript enetitled " **Positron emission tomography tracers for synucleinopathies**" (MOND-D-24-00525). You indicated that it is potentially acceptable for publication in Molecular Neurodegeneration, once we have carried out some essential revisions suggested bythe reviewers.

We have now carefully digested the comments from 2 referees, and addressed the raised concerns in the revised review article. The revised parts are highlighted in RED.

We trust the revised manuscript is now appropriate for acceptance for publication!

Best Regards,

Keqiang Ye, Ph.D.

Head, Department of Biology

Faculty of Life & Health Sciences

SIAT, Shenzhen, China
